# Wetland salinity induces sex-dependent carry-over effects on the individual performance of a long-distance migrant

**DOI:** 10.1038/s41598-017-07258-w

**Published:** 2017-07-31

**Authors:** José A. Masero, José M. Abad-Gómez, Jorge S. Gutiérrez, Francisco Santiago-Quesada, Nathan R. Senner, Juan M. Sánchez-Guzmán, Theunis Piersma, Julia Schroeder, Juan A. Amat, Auxiliadora Villegas

**Affiliations:** 1 Conservation Biology Research Group, Department of Anatomy, Cell Biology and Zoology, Faculty of Sciences, University of Extremadura, 06006 Badajoz, Spain; 2 NIOZ Royal Netherlands Institute for Sea Research, Department of Coastal Systems and Utrecht University, PO Box 59, 1790 AB Den Burg, Texel The Netherlands; 3 Conservation Ecology Group, Groningen Institute for Evolutionary Life Sciences (GELIFES), University of Groningen, PO Box 11103, 9700 CC Groningen, The Netherlands; 4 Imperial College London, Department of Life Sciences, Silwood Park Campus, London, United Kingdom; 5 Department of Wetland Ecology, Doñana Biological Station (EBD-CSIC), 41092 Seville, Spain; 6 Present Address: Division of Biological Sciences, University of Montana, 32 Campus Drive, Missoula, Montana 59802 USA

## Abstract

Salinization is having a major impact on wetlands and its biota worldwide. Specifically, many migratory animals that rely on wetlands are increasingly exposed to elevated salinity on their nonbreeding grounds. Experimental evidence suggests that physiological challenges associated with increasing salinity may disrupt self-maintenance processes in these species. Nonetheless, the potential role of salinity as a driver of ecological carry-over effects remains unstudied. Here, we investigated the extent to which the use of saline wetlands during winter – inferred from feather stable isotope values – induces residual effects that carry over and influence physiological traits relevant to fitness in black-tailed godwits *Limosa limosa limosa* on their northward migration. Overwintering males and females were segregated by wetland salinity in West Africa, with females mostly occupying freshwater wetlands. The use of these wetlands along a gradient of salinities was associated with differences in immune responsiveness to phytohaemagglutinin and sized-corrected body mass in godwits staging in southern Europe during northward migration – 3,000 km from the nonbreeding grounds – but in males only. These findings provide a window onto the processes by which wetland salinity can induce carry-over effects and can help predict how migratory species should respond to future climate-induced increases in salinity.

## Introduction

Salinization has been identified as a widespread threat to inland and coastal wetlands worldwide^1^. More than one-fourth of the world’s wetlands have been already lost due to salinization, while many others are currently threatened by this process^2^. Direct human alterations, often in conjunction with climate change, are expected to exacerbate this critical situation, meaning that the impact of wetland salinization on wildlife is currently a conservation issue of global concern^1, 3^.

Many species of vertebrates perform annual migrations or dispersions during which they are subject to large changes in the salinity of their environments^4^. In migratory animals that move regularly between freshwater and saline wetlands, recent experimental data have shown that osmoregulation is energetically expensive, and that the strength of an individual’s immune response and body mass are negatively influenced by high salinities^4, 5^. Remarkably, this experimental evidence was obtained from dunlin *Calidris alpina*, a long-distance migratory waterbird that predominantly overwinters in coastal saline wetlands. Thus, the salinity levels experienced by an individual are not without costs, even for a species well equipped to deal with saline environments^6^.

There is growing evidence that winter habitat quality can induce carry-over effects that influence individual fitness in subsequent seasons^7–9^. These carry-over effects are thought to be a common phenomenon across taxa, and may be especially exacerbated in the case of long-distance migratory species^10^. In an ecological context, the potential existence of carry-over effects makes it difficult to directly connect population dynamics with local ecological conditions, and ultimately, to guide conservation efforts^9^. Carry-over effects should therefore be considered explicitly in threat assessments and conservation planning in order to understand the long-term consequences of environmental stressors^11^.

Given that high salinity levels may disrupt physiological processes that are critical for fitness^3, 5^, it is reasonable to predict that the use of saline wetlands during the nonbreeding season may induce residual effects that carry over and influence fitness-relevant changes in performance during subsequent portions of the annual cycle. However, despite a substantial and growing amount of research focused on how salinization impacts the structure, composition and survival of wetland biota^1, 3^, no study has examined the potential for ecological carry-over effects resulting from wetland salinity to act as a driver of fitness – or performance as a proxy of fitness – in migratory animals.

Migratory waterbirds are a key component of coastal wetland biodiversity, playing vital roles in nutrient cycling, energy flow, and seed dispersal^12, 13^. Among the earliest and potentially most severe effects of climate change on these waterbirds is the increased salinity of coastal freshwater wetlands caused by sea-level rise^14^. For instance, the coastal wetlands of West Africa — from Mauritania to Sierra Leone — support millions of migratory waterbirds during the boreal winter^15^. In this region, migratory and sedentary waterbirds are facing increasing salinization and natural habitat loss due to agricultural activities^16, 17^. Rice fields, for example, currently cover large areas, and are a key component of the coastal landscape^17^. As in other parts of the world, these rice fields are used as feeding grounds by large numbers waterbirds, and rice kernels have become a fundamental component of waterbird diet^13^. The majority of rice fields are located in two contrasting environments: freshwater rice fields and sea-influenced rice fields^16, 17^. The latter are located close to the intertidal areas, where seawater intrusion during high tide is a regular occurrence; although potentially problematic for waterbirds, this intrusion is not a problem for farmers, since flooding with seawater is a common practice to prevent acidification^16^. A large salinity gradient therefore exists across the mosaic of flooded rice fields used as foraging grounds by many migratory waterbirds.

The continental black-tailed godwit *Limosa limosa limosa* (hereafter, ‘godwits’) is a near-threatened, long-distance migratory shorebird that breeds predominantly in The Netherlands and spends the nonbreeding season in coastal and inland wetlands of West Africa^18^. As with other long-distance migrants, godwits *en route* to the breeding grounds rely on a few spring staging sites^18^, which play a critical role in their annual cycle^19^. Today the main diet of godwits in West Africa is unharvested rice kernels that they obtain by foraging in both flooded freshwater and sea-influenced rice fields^17, 20^. Because continental godwits historically only occurred in freshwater or brackish wetlands^21, 22^, they will likely be negatively affected by significant time spent in highly saline environments.

We investigated the extent to which the use of saline wetlands during the nonbreeding season induce residual effects that carry over and influence physiological traits relevant to fitness in black-tailed godwits as they return to their breeding grounds. Specifically, we examined whether variation in winter wetland salinity – inferred from feather stable isotope values – resulted in systematic variation in both the ability to mount a phytohaemagglutinin-induced pro-inflammatory immune response (PHA-induced response) and size-corrected body mass in godwits staging at Extremadura, Spain, a major European spring staging site located some thousands of kilometers away from the main African nonbreeding sites^18, 23^. Both physiological performance traits are frequently correlated with fitness: the PHA-induced response has been associated with longevity and survival in several migratory bird species^24^, while size-corrected body mass is a body condition index related to an individual’s health, quality and vigor^25^. Both PHA-induced immune response and size-corrected body mass are therefore potentially useful physiological traits with which to detect immunological and energetic carry-over effects, respectively, of habitat occupancy from the nonbreeding season to subsequent time periods in migratory species. The two physiological traits are not necessarily correlated with each other, and separate studies have reported that size-corrected body mass does not influence the intensity of the PHA-induced response^26^. In this context, an individual’s immunocompetence at the end of one season may represent preparations for subsequent seasons^7^, but the potential for carry-over effects from nonbreeding habitats to affect immune defense in the future remains unexplored.

Sex is another major, largely unstudied, source of potential variation in carry-over effects. Recently, sex-specific carry-over effects have been shown to contribute to population declines in a long-distance migratory population of barn swallows *Hirundo rustica* ^27^. Godwits are strongly sexually dimorphic in size and breeding plumage coloration, with females being larger and duller plumaged^19^. As such, sex may promote segregation in some black-tailed godwit populations, as females are the dominant sex outside the breeding^28^. Here, using salinity as an indicator of habitat quality, we hypothesized that male and female godwits would be segregated on their African nonbreeding grounds and that females would predominate in rice fields located in freshwater environments. We predicted that the use of saline wetlands would contribute to the generation of sex-related and inter-individual differences in immune responsiveness and/or size-corrected body mass, and that such differences would still be evident at spring staging sites.

## Results

**Figure 1:**
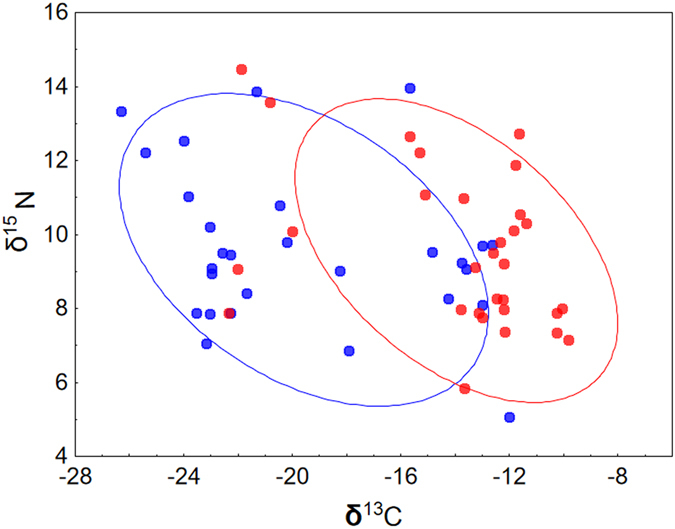
Stable carbon and nitrogen isotope values in primary feathers (P10) of male () and female () black-tailed godwits staging in Extremadura rice fields during northward migration. Gaussian bivariate ellipses (95% probability interval of the mean population) are shown.

Carbon (δ^13^C) feather values ranged from −26.32‰ to −5.06‰, indicating that overwintering godwits used a diversity of wetlands, from freshwater to saline habitats. Sex was significantly associated with these δ^13^C feather values, and mean values in males were higher than in females (Table 1, Fig. 1). Nitrogen (δ^15^N) feather values ranged from 5.06‰ to 14.48‰, but did not differ between sexes (Table 1, Fig. 1).

**Table 1:** Mean values (±SE and 95% confident intervals) of PHA-induced response (PHA), body condition (SMI), and carbon (δ^13^C) and nitrogen (δ^15^N) stable isotopes in male and female black-tailed godwits staging in Extremadura.

	males	females	*F*	*P*
PHA (mm)*	0.70 ± 0.08 (0.54–0.87)	0.32 ± 0.10 (0.12–0.52)	*F*_1,17_ = 7.33	0.015
SMI (g)**	296.38 ± 7.56 (281.23–311.52)	323.39 ± 7.81 (307.73–339.05)	*F*_1,55_ = 5.86	0.017
δ^13^C***	−14.37 ± 0.77 (−15.91–−12.94)	−19.13 ± 0.79 (−20.72–−17.54)	*F*_1,55_ = 17.22	<0.0001
δ^15^N***	9.64 ± 0.41 (8.84–10.46)	9.51 ± 0.42 (8.65–10.33)	*F*_1,55_ = 0.04	0.83

See text for sample sizes and statistical details. ^*^Model including body mass^1/3^ as covariate (*F*_1,55_ = 1.18, *P* = 0.29). **The reduced model only included sex as fixed factor and date as covariate (*F*_1,55_ = 8.00, *P* < 0.01). ***The model included SMI as covariate (*P* > 0.05 in both cases).

**Figure 2:**
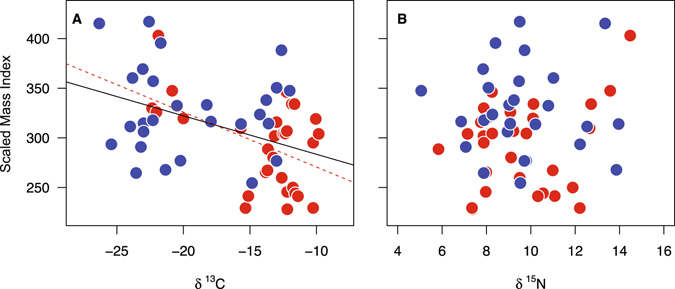
Stable carbon and nitrogen values of primary feathers (P10) moulted in Africa in relation to their size-corrected body mass (Scaled Mass Index; g) in the European staging site. (): males, (): females. Regression lines are only shown for significant relationships (solid line: males and females combined; dotted line: males).

There was a negative and statistically significant relationship between δ^13^C feather values and scaled mass index (SMI) at the spring staging site (*r* = −0.42, *P* < 0.005, *n* = 58; Fig. 2A), with individuals from more freshwater wetlands showing the greatest SMI values. When considering each sex separately, this relationship was statistically significant for males (*r* = −0.48, *P* < 0.01, *n* = 30; Fig. 2A), but not females (*r* = −0.08, *P* = 0.67, *n* = 28; Fig. 2A). δ^15^N values and SMI were not associated with each other (overall: *r* = 0.24, *P* = 0.20, *n* = 58, Fig. 2B; males: *r* = −0.04, *P* = 0.84, *n* = 30, Fig. 2B; females: *r* = 0.10, *P* = 0.46, *n* = 28, Fig. 2B).

**Figure 3:**
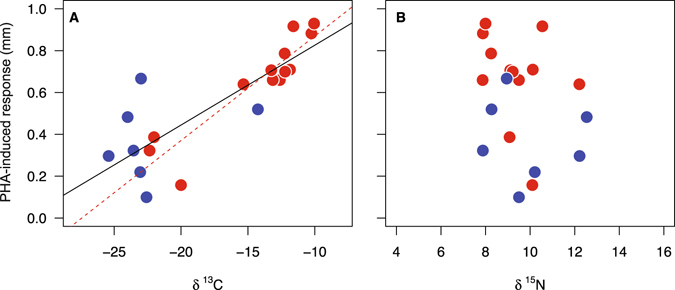
Immune responsiveness to PHA of black-tailed godwits in the European staging site in relation to stable carbon and nitrogen values of primary feathers moulted previously in Africa. (): males, (): females. Regression lines are only shown for significant relationships (solid line: males and females combined; dotted line: males).

Overall, the PHA-induced response at the spring staging site was significantly and positively correlated with δ^13^C feather values (*r* = 0.83, *P* < 0.001, *n* = 20; Fig. 3A). Analysis of each sex separately revealed a significant positive relationship between PHA-induced response and δ^13^C feather values in males (*r* = 0.91, *P* < 0.001, *n* = 13; Fig. 3A), but not females (*r* = 0.30, *P* = 0.51, *n* = 7; Fig. 3A). δ^15^N feather values and PHA-induced response, however, were not correlated (overall: *r* = 0.21, *P* = 0.36, *n* = 20; Fig. 3B; males: *r* = 0.10, *P* = 0.73, *n* = 13; Fig. 3B; females: *r* = 0.11, *P* = 0.80, *n* = 7; Fig. 3B).

The PHA-induced response was higher in males than females (Table 1). These differences were statistically significant both with body mass^1/3^ (Table 1) or SMI as the predictive variable (Sex: *F*_1,17_ = 5.62, *P* < 0.05; SMI: *F*_1,17_ = 1.97, *P* = 0.18). SMI was higher in females than males (Table 1).

The most well supported models explaining variation in SMI and the PHA-induced response of males at the spring staging site included the δ^13^C values of African feathers (Table 2). This predictor was present in all models with a difference in Akaike’s information criterion corrected for small sample size (ΔAICc) <2 and best explained the observed variation (sum of Akaike’s weights ranging from 0.94 to 1.00, Table 2). In females, the best supported model explaining SMI included Julian date (Table 2); Julian date was present in all explanatory models within ΔAICc <2 and had a relative importance (RI) of 0.92, as compared to 0.40 for δ^13^C.

**Table 2:** Explanatory models of (a) size-corrected body mass (Scaled Mass Index) and (b) PHA-induced of godwits, selected according to Akaike’s information criterion adjusted for small samples (AICc).

Model rank	Variables	−2Log*L*	*K*	AICc	ΔAICc	*w*_*i*_
(a) ***Scaled Mass Index***						
Males (n = 30)						
1	*δ* ^*13*^ *C* *+* *date*	299.56	4	305.56	0.00	0.14
2	*δ* ^*13*^ *C*	301.63	3	305.63	0.06	0.13
3	*δ* ^*13*^ *C* *+* *BP*	300.40	4	306.40	0.84	0.09
4	*δ* ^*13*^ *C* *+* *date* *+* *year*	298.54	5	306.54	0.98	0.08
5	*δ* ^*13*^ *C* *+* *date* *+* *BP*	298.90	5	306.90	1.33	0.07
6	*δ* ^*13*^ *C* *+* *date* *+* *δ* ^*15*^ *N*	299.27	5	307.27	1.70	0.06
7	*δ* ^*13*^ *C* *+* *δ* ^*15*^ *N*	301.40	4	307.40	1.84	0.05
8	*δ* ^*13*^ *C* *+* *year*	301.43	4	307.43	1.87	0.05
9	*Null model*		2			
Females (*n* = 28)						
1	*date*	283.21	3	287.21	0.00	0.19
2	*date* *+* *δ* ^*13*^ *C*	282.00	4	288.00	0.80	0.13
3	*date* *+* *BP*	282.88	4	288.88	1.67	0.08
4	*date* *+* *δ* ^*15*^ *N*	282.95	4	288.95	1.74	0.08
5	*date* *+* *year*	283.00	4	289.00	1.80	0.08
6	*date* *+* *δ* ^*13*^ *C* *+* *δ* ^*15*^ *N*	281.12	5	289.12	1.92	0.07
7	*Null model*		2			
(b) ***PHA-immune response***						
Males (n = 13)						
	*δ* ^*13*^ *C*	−28.43	3	−24.43	0.00	0.27
	*δ* ^*13*^ *C* *+* *SMI*	−30.04	4	−24.04	0.39	0.23
	*δ* ^*13*^ *C* *+* *δ* ^*15*^ *N*	−28.91	4	−22.91	1.52	0.13
	*δ* ^*13*^ *C* *+* *BP*	−28.52	4	−22.52	1.91	0.11
	*Null model*					

Only candidate models with ∆AICc < 2 and the null model are ranked according to AICc weights (*w*_*i*_). *K*: the number of parameters in the model (including intercept and variance); BP: breeding plumage score; date: Julian date. Parameter Akaike weights: (a) Males: δ^13^C = 0.94, date = 0.53, BP: 0.37, year = 0.36, δ^15^N = 0.27. Females: date = 0.92, δ^13^C = 0.40, BP: 0.35, δ^15^N = 0.31, year = 0.29; (b) Males: δ^13^C = 1.00, SMI = 0.44, δ^15^N = 0.30, BP = 0.29.

## Discussion

By combining measures of feather stable isotope values to infer nonbreeding habitat use and physiological traits relevant to fitness in a long-distance migrating bird, we show for the first time that salinity levels can induce sex-dependent carry-over effects that persist several thousand kilometers from the nonbreeding grounds. The isotopic composition of feathers indicate that godwits staging in southern Europe during northward migration occupied wetlands along a broad salinity gradient during the previous nonbreeding season. The salinity of these wetlands was linked to variation in body condition and the ability to mount a pro-inflammatory immune response several weeks after leaving their African nonbreeding sites. However, the salinity of African wetlands was linked to both physiological traits at the spring staging site in males only, suggesting that such carry-over effects of wetland salinity may impact each sex differently. To the best of our knowledge, this is the first study supporting the existence of ecological carry-over effects driven by wetland salinity on physiological traits in a long-distance migratory animal.

Large-scale surveys of the godwit nonbreeding range in West Africa have shown that most individuals rely on either sea-influenced or freshwater rice fields^20^, with negligible godwit densities in the other habitats^12^. The higher δ^13^C values found in males likely reflect the fact that they predominantly use sea-influenced rice fields, while the vast majority of females showed more negative δ^13^C values, suggesting that they make more extensive use of freshwater rice fields. This pattern mirrors that of a previous study using δ^13^C values in body feathers of Dutch-breeding godwits from the breeding grounds, which showed that males had on average higher δ^13^C values than females, but that there was no difference in δ^15^N values between the sexes^22^. Therefore, the greater use of freshwater and sea-influenced rice fields by wintering female and male godwits, respectively, appears to be a consistent pattern.

The energetic costs associated with osmoregulation may significantly affect energy budgets and the resulting habitat choices in birds^29, 30^, teleost fishes^31, 32^, amphibians^3, 33^, and reptiles^34, 35^. By choosing freshwater rice fields, female godwits likely reduce the energetic costs associated with osmoregulation. Thus, from this perspective, females may be ‘monopolizing’ the best quality foraging grounds. Previous studies on other shorebird species — including Icelandic black-tailed godwits *L. l. islandica* ^28^, bar-tailed godwits *L. lapponica* ^36, 37^, and red knots *Calidris canutus* ^38^ — found evidence for sexual segregation outside the breeding season, with females thought to be the dominant sex^36, 37^. Our observations during several experiments performed with godwits in captivity^39^, including those carried out in this study, are in accordance with this suggestion: we have found that captive females initiate and win most (>90%) aggressive intersexual encounters (J. A. Masero *et al*., unpublished data).

Moult is one of the most energy-demanding and time-consuming events in the annual cycle of long-distance migratory birds, and most avian species separate moult from other energetically-demanding activities^40, 41^. Accordingly, the basal metabolic rate of long-distance migratory shorebirds has been shown to increase by 10% during primary feather moult^42^. Our experiment suggests that godwits using Extremadura as spring staging site moulted their flight feathers in West Africa during early winter. Later, prior to leaving West Africa, many godwits also begin to undergo the transition from nonbreeding to breeding plumage, with males being the more colourful sex and thus required to produce more costly pigmented feathers^43^.

At the same time, osmoregulation entails a substantial increase in energy expenditure and metabolic rates in birds^6, 44^. For example, basal metabolic rate increased by 17% and daily energy consumption increased by 20% in freshwater-acclimated dunlin after exposure to seawater^4^. Although the timing and extent of trade-offs between osmoregulation and other energy-demanding activities are poorly understood^6^, recent data have revealed that the simultaneous demands of osmoregulation and energetically costly activities such as thermoregulation significantly reduce condition-related traits, while increasing stress related ones^45^. Furthermore, before leaving the wintering grounds, godwits, as with most long-distance migrants, face the challenge of generating the large fuel stores required for migration. In the case of godwits occupying saline wetlands, they must therefore overlap the costs of fuel deposition prior to migration (shorebirds may double their pre-migration body mass^46^) with those of osmoregulation, potentially leading to energetic constraints. Overwintering godwits in saline wetlands, therefore, face potential energetic trade-offs between osmoregulation and moult, and between osmoregulation and pre-migratory hyperphagia, making godwits from saline wetlands more susceptible to ecological carry-over effects from the nonbreeding season to successive life history stages.

Indeed, males that used more saline wetlands in winter arrived at staging sites in Extremadura in poorer body condition than males that used freshwater wetlands. It is generally assumed that migrants arriving at stopover or staging sites with low body reserves stay longer than those that arrive at the same sites with higher stores^47^. However, a growing number of studies have failed to confirm a relationship between the length of stay and body condition at departure, suggesting that spatiotemporal programmes may play an important role in controlling stopover duration^48^. Data from radio-tagged godwits using Extremadura rice fields during northward migration^21^ support these results and suggest that body condition at capture is also not related to the length of stay in this spring staging site in godwits (11 males and 13 females; *r* ^*2*^ < 0.15, *P* > 0.12 for both sexes). Additionally, departure from Iberian staging sites, arrival to the breeding grounds, and egg laying date are moderately repeatable in individual male and female godwits across years^49^. If males from saline wetlands also arrive at the breeding areas in poorer body condition than males from freshwater wetlands because they have an endogenous migratory schedule that controls their migratory timing and constrains them from staying at staging sites longer to improve their body condition, carry-over effects of nonbreeding salinity could create fitness inequalities among male godwits.

We found that male godwits from saline wetlands exhibited a lower body condition index, but a higher responsiveness to PHA. In small migratory passerines, the production of a strong inflammatory response to PHA have been associated with a general fitness advantage^24^. In godwits, however, the benefits of high immune responsiveness may be counter-balanced if the lower size-corrected body mass associated with the use of saline wetlands reduces fitness. In addition, in some bird species, a greater inflammatory swelling caused by responsiveness to PHA indicates lower, rather than higher individual quality^50^. In combination, then, the association between feather δ^13^C values and an individuals’ pro-inflammatory potential in Extremadura suggests that environmental salinity experienced by overwintering godwits in Africa may be a determinant of their ability to physiologically react to infections at freshwater sites located several thousands of kilometers apart, some weeks after departing from wintering sites. This is crucial when considering the potential fitness effects of salinity on long-distance migrants.

Many waterbirds, as well as some passerines, can cope successfully with a diet that imposes high osmotic loads^51, 52^. For instance, the ability of some waterbirds to inhabit saline wetlands has been attributed to a suite of behavioural, anatomical, and physiological adaptations to either eliminate excess salt or to reduce the intake of saline water by selecting diets with high ‘free water’ contents to compensate for their limited physiological capabilities^6^. However, many other migratory species less well-equipped to deal with saline environments are increasingly exposed to elevated salinity in their non-breeding habitats owing to global change^4^, presenting physiological challenges. We found that the use of wetlands in West Africa along a gradient of salinities was associated with differences in immune responsiveness and body condition in godwits at a key staging site in southern Europe. These findings provide a window onto the processes by which habitat quality associated to wetland salinity could potentially influence population processes through sex-dependent carry-over effects, and how migratory species may respond to future environmental changes^6^.

## Methods

**Figure 4:**
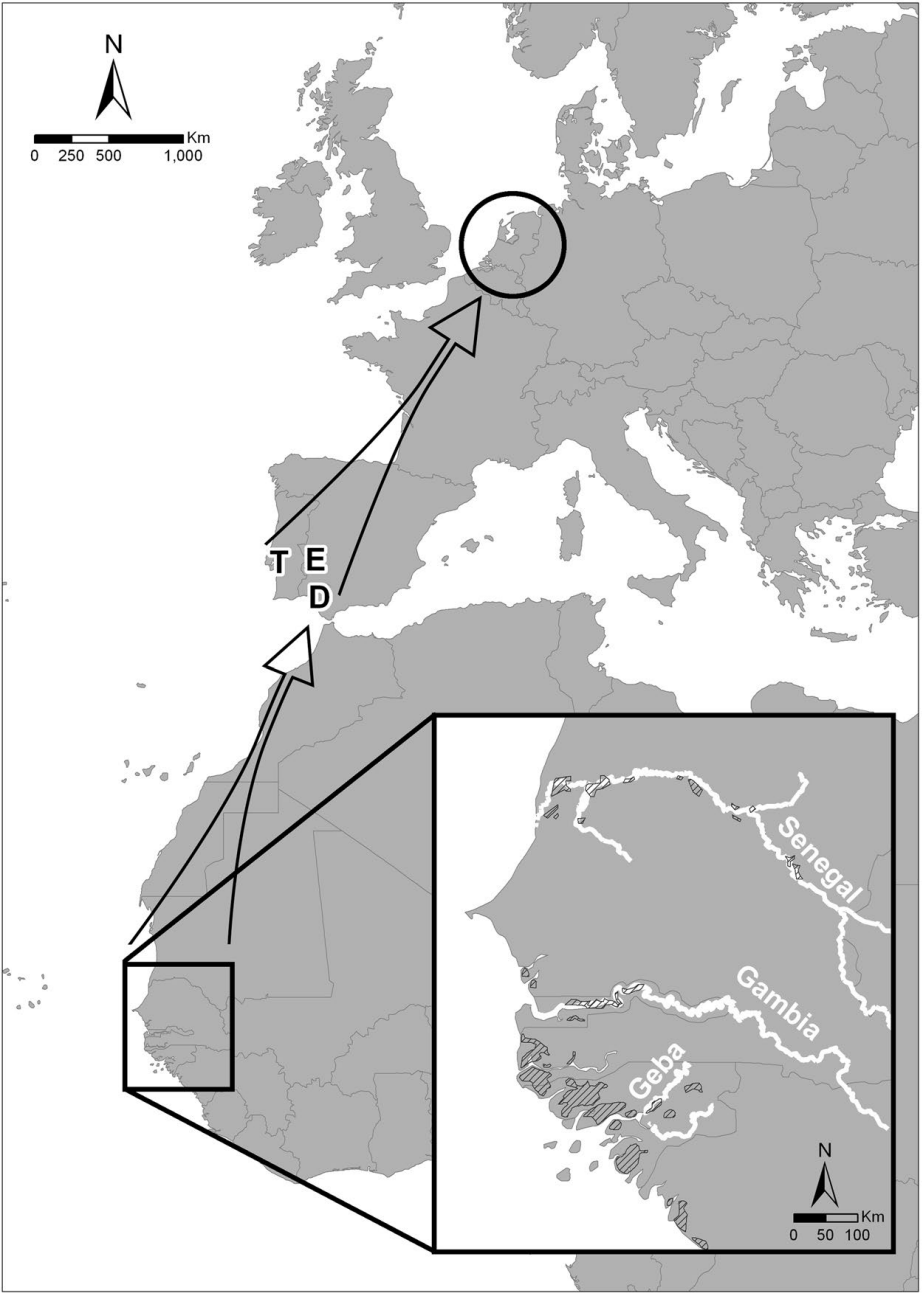
Map showing the location of main breeding (Western Europe) and wintering (West Africa) grounds of continental black-tailed godwits *Limosa limosa limosa*. During northward migration, most individuals fly non-stop from wetlands in West Africa to their major staging sites in southern Europe (D: Doñana, T: Tagus estuary, E: Extremadura rice fields). Arrows show direction of major migration routes, and lined areas show the main rice fields used by godwits during the non-breeding season in West Africa^20^. Map was created with ArcGIS version 10.1 (http://www.arcgis.com/).

Data from ringing recoveries and satellite transmitters indicate that most godwits breeding in Western Europe winter in West Africa, mainly in rice fields from southern Senegal to Guinea Bissau, as well as in the large Sahelian floodplains of the Senegal and Niger rivers^17, 18^ (see Fig. 4). Subsequently, most godwits that leave West Africa for their breeding grounds in northwestern Europe stop in rice fields in SW Iberia^18, 23^. Our study was performed at the rice fields of Extremadura, Spain (see Fig. 4). During northward migration (late January-early March), these rice fields are a key staging site for migrating godwits, with at least 14% of the flyway population stopping there during the period from 2005–2011^23^. Birds arrive at Extremadura in late January, with peak migration occurring in early-to-mid February, and most birds staying for 2–5 weeks^18, 23^.

Data from godwits captured in Extremadura and equipped with satellite transmitters showed that most godwits do not arrive directly to Extremadura after leaving West Africa. In 2013 and 2014 we placed satellite transmitters on 30 godwits in Extremadura^18^. Of those 30 individuals, we then tracked 17 for their return northward migration the next year. Ten of those 17 individuals, again staged in Extremadura, nine of which first stopped in either Doñana or the Tagus estuary (see Fig. 4) for an average of 26.78 ± 15.47 days (range = 8–50 days). There are no sex-related differences in the timing of migration.

### Scaled Mass Index

During the peak of migration, we captured godwits (30 males and 28 females; years 2007, 2008 and 2011) using mist nets placed near roosting areas in central Extremadura^23^ (39.0364°N, 5.9112°W). All birds were individually colour-marked, measured (culmen and tarsus length, accuracy ±0.01 mm; wing length both flattened and straightened, ±0.5 mm), weighed (±0.01 g), and aged according to plumage characteristics. Overwintering godwits undergo a seasonal colour change from non-breeding to breeding plumage. We scored the extent of the breeding plumage as a percentage (5% increments) of the expected appearance of fully moulted male and female godwits^53^, based on an assessment of both under- and upperparts^21^. We first assigned sex to each individual according its relative bill length, and later confirmed this through molecular sexing from blood samples extracted from the brachial vein^39^. A few individuals incorrectly sexed according to bill length were discarded from the analyses, since their breeding plumage was scored erroneously. Finally, we used DNA analyses of these blood samples to ensure that only birds of the *limosa* subspecies were included in our analyses^54^.

Body condition was assessed on the basis of the SMI (g), a method that normalizes body mass to a fixed value of body size based on the scaling relationship between mass and length measures^55^, and has been shown to perform better than other potential body condition indices^25^. Because godwits are sexually dimorphic in size^56^, we calculated SMI separately for males and females. The fixed value of body size was the mean value of wing length for the study population: 223.35 ± 0.53 mm (n = 147) for females and 212.67 ± 0.40 mm (n = 218) for males (data from adult godwits caught in Extremadura from 2004–2012).

### Stable isotope analysis

We collected a small fragment from the distal tip of an outer primary feather (P10) for stable isotope analysis. Measurements of δ^13^C and δ^15^N stable isotopes in bird tissues can be used to delineate habitat use and dietary-trophic levels, respectively^57^. Specifically, more negative δ^13^C values indicate a high degree of use of freshwater habitats, while more positive δ^13^C values indicate marine habitat use^58^. Overall, δ^13^C feather and blood values of non-breeding waterbirds, including godwits, become more positive with increasing salinity^58–60^. Accordingly, previous studies have found that within a single coastal system with a diversity of wetlands, ranging from freshwater to hypersaline habitats, δ^13^C signatures in blood and feathers of waterbirds accurately reflect the habitat use of individuals according to wetland salinity^60^. In the case of godwits, the δ^13^C values in whole blood samples of individuals feeding exclusively on freshwater rice fields in Extremadura were <−21‰^13^, while the δ^13^C values in individuals feeding on macroinvertebrates in marine environments at Cadiz Bay (SW Spain) were >−17‰ (*n* = 7; J.A. Masero unpubl. data).

Most overwintering godwits feed on a single food type — rice kernels left on the ground after harvest — which they obtain by feeding in flooded rice fields. Unfortunately, the isotopic signature of rice seeds from the region of West Africa inhabited by godwits in this study were not available. Furthermore, because rice is a C3 plant that uses atmospheric CO_2_, we would expect it to exhibit a similar isotopic signature independent of whether it grows in salt- or freshwater environments. However, the carbon isotopic signature of C3 plants – including rice – may be modified by salinity, and it has been shown that the δ^13^C values of rice are generally more positive as salinity increases^61^. Additionally, and more importantly, we noted during studies of foraging behaviour that godwits drink water directly from the water column of flooded rice fields and saltpans^62^. After harvesting, coastal rice fields are flooded, intentionally or not, with seawater (see Introduction), meaning that godwits using these coastal rice fields must drink saline water and thereby increase their salt loads. As the δ^13^C values of seawater (dissolved and particulate organic carbon) are more positive than those of their freshwater counterparts^63^, we are confident that godwits drinking and eating in coastal rice fields flooded with saline water have more positive δ^13^C values in their primary feathers than those birds that moulted their primary feathers while using rice fields flooded with freshwater.

Feathers integrate isotopic information over the period during which they were grown. As the primary moult chronology of continental godwits is poorly known, we performed a study to document the pattern of primary moult in the subspecies. A cohort of 20 adult godwits captured during northward migration (10 males and 10 females) was maintained in captivity until primary moult was completed the following fall. Once primary moult began, wings were examined regularly (1–2 times per week) to determine the timing and duration of P10 moult. In both sexes, P10 emerged between mid-November and mid-December, i.e. coinciding with the nonbreeding period in West Africa (J.A. Masero unpubl. data). We can thus safely assume that the feather samples we collected in Extremadura during northward migration were grown in West Africa.

Stable-isotope analyses were performed at the Iso-Analytical Laboratory (Cheshire, UK). Stable isotope ratios were reported using the typical δ-notation relative to international standards (Vienna PeeDee Belemnite for C, and atmospheric nitrogen for N) in parts per thousand (for example, δ^13^C = (^13^C/^12^C[sample]/^13^C/^12^C[standard] − 1) × 1000).

### Immunological assessment

To estimate the PHA-induced response, after collecting the distal tip of P10 (see above) and body measurements, a random subset of 20 individuals (molecular sexing: 13 males and 7 females) captured on 15 February 2011 was immediately transported to the University of Extremadura where they were housed in large outdoor aviaries (4–6 individuals per aviary; males and females mixed). Each aviary was equipped with a drinking/bathing pool (2.5 × 2 m; 5 cm depth), and the roof and sides of the aviary were covered with a tiny, soft mesh to avoid potential injuries during flights inside the aviary. Prior to the PHA test, birds were acclimatized for three days to captivity. During this period, as well as throughout the experiment, godwits had access to freshwater for drinking, bathing, and feeding (rice seeds, fly larvae *Protophormia terraenovae*, and commercial food pellets) *ad libitum*.

We injected 60 µl of 1 mg ml^−1^ PHA-P (Sigma L-8754) in phosphate-buffered saline (PBS) intra-dermally into one wing web of each godwit. This volume and dosage is known to work in shorebirds^5^. We measured the thickness (accuracy ± 0.01 mm) of the injected wing web in triplicate with a pressure-sensitive digital spessimeter (Mitutoyo Absolute cod. 543-162 270BS, Japan) prior to injection and again after 24 h. The repeatability of measurements was very high (r = 0.99, p < 0.001), so we used the mean value for statistical analyses. The inflammatory response (swelling of the wing web) was calculated as the difference in thickness of the wing web prior to and after the PHA injection.

### Statistical analyses

We used linear correlations to test for the relationships between isotopic values and the two physiological traits, both overall and separately for each sex. We used a general linear model (GLM) to test for the effect of sex on SMI, using sex, year, and a year × sex interaction term as fixed factors. Breeding plumage score and Julian date were included as covariates, since both variables can predict body condition in shorebirds^64^. Breeding plumage score data were arcsin-transformed to meet model assumptions. To obtain a reduced model, we carried out a stepwise backwards elimination by sequentially deleting terms with a *P*-value > 0.05, starting with the interaction term.

We performed another GLM to test for the effect of sex on the strength of PHA-induced response, with SMI as covariate, because immune response can be condition-dependent^65^. Sex-related differences in the strength of PHA-induced response can be the result of different relative dosages, given that all godwits were given the same dosage, but females are larger than males. Therefore, potential sex-related differences in the PHA-induced response were also assessed by including ‘body mass^1/3^’ as covariate^66^. Differences between the sexes in stable isotope levels were assessed using GLMs that included sex and SMI as a fixed factor and a covariate, respectively.

The factors affecting SMI were analyzed in males and females separately, and candidate models (n = 31 for each sex) were built using all combinations of the predictors δ^13^C, δ^15^N, breeding plumage score, Julian date, and year. To avoid over-parameterization given the small sample size, we excluded interaction terms^67^. The factors affecting the PHA-induced response were only analyzed in males owing to an insufficient sample size for females (n = 7); candidate models (n = 16) were built using all combinations (but excluding interactions; see above) of the predictors, δ^13^C, δ^15^N, SMI, and breeding plumage score. The explanatory variables included in the models were not highly correlated with each other (r < 0.5). Models were ranked according to AIC_c_^68^. A ∆AIC_c_ score relative to the minimum-AIC_c_ model was computed for each model. From the ΔAIC_c_, we computed Akaike model weights. We calculated the RI of each explanatory variable by calculating the sum of the Akaike weights of the models that contained that variable. We report means ± standard error and 95% confidence intervals. All analyses were conducted using R (v. 3.1.0, R Core Development Team 2014) and SPSS 19.0.

### Ethic statement

This study conforms to Spanish legal requirements, and was conducted under authorization from Government of Extremadura (CN04/1224). The experimental protocol was approved by the University of Extremadura.

### Data availability

All data generated or analysed during this study are included in this published article (Supplementary Information file).

## Electronic supplementary material

Dataset1
